# Sex differences in correlates of suicide attempts in Chinese Han first‐episode and drug‐naïve major depressive disorder with comorbid subclinical hypothyroidism: A cross‐sectional study

**DOI:** 10.1002/brb3.3578

**Published:** 2024-06-06

**Authors:** Xue Tian, Xiao‐En Liu, Fengfeng Bai, Meijuan Li, Yuying Qiu, Qingyan Jiao, Jie Li, Xiang‐Yang Zhang

**Affiliations:** ^1^ Institute of Mental Health Tianjin Anding Hospital, Mental Health Center of Tianjin Medical University Tianjin China; ^2^ CAS Key Laboratory of Mental Health Institute of Psychology Chinese Academy of Sciences Beijing China; ^3^ Department of Psychology University of Chinese Academy of Sciences Beijing China

**Keywords:** major depressive disorder, sex differences, subclinical hypothyroidism, suicide

## Abstract

**Background:**

This study aimed to investigate sex differences in risk factors for suicide attempts in first‐episode and drug naive (FEDN) major depressive disorder (MDD) with comorbid subclinical hypothyroidism (SCH).

**Methods:**

A total of 1034 FEDN MDD patients with comorbid SCH were enrolled. The Hamilton Depression Scale (HAMD), Hamilton Anxiety Scale (HAMA), and Positive and Negative Syndrome Scale (PANSS) positive subscale were used to assess patients’ symptoms. Thyroid hormone levels and metabolic parameters were measured.

**Results:**

MDD patients with SCH had a significantly higher risk of suicide attempts than those without SCH (25.4% vs. 12.2%). Logistic regression showed that HAMA score, thyroid stimulating hormone (TSH) levels, and thyroid peroxidase antibody (TPOAb) levels were significantly associated with an increased risk for suicide attempts in both male and female MDD patients comorbid SCH, while low‐density lipoprotein cholesterol (LDL‐C) was significantly associated with an increased risk for suicide attempts only in male patients, HAMD score and systolic blood pressure were significantly associated with an increased risk for suicide attempts only in female patients.

**Conclusion:**

SCH comorbidities may increase suicide attempts in MDD patients. Our results showed significant sex differences in clinical and metabolic factors associated with suicide attempts among FEDN MDD patients with comorbid SCH, highlighting appropriate sex‐based preventive interventions are needed.

## Graphical abstract

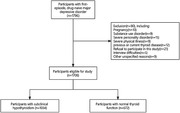

## 1 INTRODUCTION

Depression, as a chronic and recurring disorder, markedly contributes to the global burden of disease (Ferrari et al., 2013). In China, depression has held the position of the second leading cause of disability since 2010 (Yang et al., 2013). The relationship between depression and alterations in the hypothalamic‐pituitary‐thyroid (HPT) axis has been well documented (Hage & Azar, 2012; Medici et al., 2014; Williams et al., 2009). Research indicates that individuals with subclinical hypothyroidism (SCH) have double the likelihood of developing depressive symptoms compared to healthy individuals (Kim et al., 2020). In addition, numerous studies have observed an increased occurrence of SCH among individuals with major depressive disorder (MDD) in comparison to the overall population (Haggerty & Prange, 1995; Wu et al., 2013). In adolescents, the prevalence of SCH among those experiencing depression stands at 9.1%, significantly higher than that observed in the general pediatric population (Hirtz et al., 2021). Recent meta‐analyses have solidified the relationship between depression and SCH (Bode et al., 2021; Samuels, 2018). The cooccurrence of SCH may exacerbate symptoms of depression. Patients with refractory depression have a higher probability of experiencing comorbid SCH in comparison to patients with generalized depression (Feldman et al., 2013). Our prior study also indicated that the cooccurrence of SCH could potentially elevate the likelihood of suicide attempts in individuals with MDD (Shangguan et al., 2022). Thus, a comprehensive exploration of the clinical characteristics of MDD accompanied by SCH holds significant importance for guiding treatment decisions.

Suicide stands as one of the most critical global health concerns. Individuals experiencing depression face a higher risk of death by suicide (15‐30%) compared to healthy individuals (Marneros, 2006; Marneros, 2009). A recent meta‐analysis showed that the lifetime incidence of suicide attempts among MDD patients was 31% (Dong et al., 2019), with a lifetime pooled incidence in China specifically at 23.7% (Dong et al., 2018). Hawton et al. (2013) represented a systematic review that identified various risk factors for suicide attempts in individuals with MDD, including sex, family history of psychiatric disorders, prior suicide attempts, heightened depression severity, feelings of hopelessness, as well as comorbidities such as anxiety, alcohol, and substance abuse. Scientists have endeavored to identify biomarkers linked to suicidal behavior and have conducted numerous studies. Studies have found that thyroid function (Jokinen et al., 2008; Peng et al., 2018; Sanna et al., 2014; Toloza et al., 2021), metabolic function (Ainiyet and Rybakowski, 2014; Baek et al., 2014; Ma et al., 2019), and blood pressure (Ge et al., 2019; Lehmann et al., 2018; Nam & Lee, 2019) are associated with suicide. However, limited research has investigated the factors contributing to suicide among MDD patients with comorbid SCH. Our previous research indicated a correlation between severe anxiety, severity of depressive symptoms, and psychotic manifestations with suicide attempts among MDD patients presenting thyroid stimulating hormone (TSH) levels above 8 mIU/L (Shangguan et al., 2022).

Depression exhibits a higher prevalence among women compared to men (GBD 2019 Mental Disorders Collaborators; Gu et al., 2013). The prevalence of thyroid disorders varies by sex, with women having a higher prevalence and older women being more likely to develop SCH (Hennessey and Espaillat, 2015; Vanderpump et al., 1995). Additionally, the relationship between depression and thyroid function is influenced by sex. A survey conducted in Korea found the potential role of sex in the relationship between TSH levels and depressive symptoms (Lee et al., 2019). Moreover, sex differences impacts the relationship between suicide and depression (Allison et al., 2001). Brådvik et al. (2008) identified males and depression severity as risk factors for suicide. Earlier studies have found that females attempt suicide significantly more often than males, while completed suicides occur more frequently among males (Miranda‐Mendizabal et al., 2019). In our previous study, we identified sex differences in the relationship between thyroid function markers and suicide attempts among young patients with MDD combined with anxiety (Ye et al., 2023).

Sex differences are important for both MDD and thyroid diseases. As far as we know, no studies have explored the sex differences in the risk factors for suicide attempts among MDD patients with SCH. This cross‐sectional study aims to investigate sex differences in (1) the prevalence of suicide attempts and (2) related factors contributing to suicide attempts among first‐episode and drug‐naïve (FEDN) MDD patients with comorbid SCH. We hypothesize that there are sex differences in the prevalence and risk factors of suicide attempts in MDD patients with comorbid SCH.

## 2 METHODS

### 2.1 Subjects

In this research, the recruitment of participants was from the Department of Psychiatry at the First Affiliated Hospital of Shanxi Medical University. The participants recruited in this study were required to meet specific criteria as follows: (1) initial diagnosis of MDD by the Diagnostic and Statistical Manual of Mental Disorders (4th edition) (DSM‐IV); (2) had not received any prior antidepressant or antipsychotic treatment; (3) were between 18 and 60 years of age, Han Chinese; (4) had a 17‐item Hamilton Rating Scale for Depression (HAMD‐17) score ≥ 24. Exclusion criteria encompassed: (1) primary diagnosis consistent with a psychiatric diagnosis other than DSM‐IV‐based MDD; (2) significant concurrent physical illness; (3) planned pregnancy, current pregnancy, or breastfeeding; (4) history of substance abuse or dependence (excluding nicotine); (5) were unable to understand the consent process or refused to provide consent; (6) had previous or current thyroid disease.

The study received approval from the Institutional Review Board (IRB) of the First Affiliated Hospital of Shanxi Medical University. The recruitment of participants adhered to the Helsinki Declaration. Written informed consent was obtained from participants or their guardians before enrollment.

### 2.2 Clinical measures

The HAMD‐17 was utilized to assess patients' depression levels (Hamilton, 1960). The 14‐item Hamilton Anxiety Rating Scale (HAMA‐14) was employed to assess the anxiety level of patients (Hamilton, 1959; Hamilton, 1959). The Chinese versions of the HAMD‐17 and HAMA‐14 have demonstrated good reliability and validity (Lin, 1986; Zhu, 1985). The positive subscale of the Positive and Negative Syndrome Scale (PANSS) was utilized to assess patients' psychotic symptoms (Kay et al., 1987).

Information on suicide attempts was gathered through face‐to‐face semistructured interviews and medical record reviews. Suicide attempts are defined as actions by an individual aimed at ending his or her life by self‐harming to some extent. Each patient was asked a question: “Have you ever attempted suicide?” Those responding “yes” were categorized as suicide attempters. Additional details were sought, such as the number of attempts, dates, and methods used. In instances of uncertainty, researchers conducted further interviews with the patient's family, close acquaintances, or clinical team to validate the information.

Two experienced psychiatrists received special training and were responsible for collecting the above information. Following multiple assessments, their interobserver correlation coefficients for HAMD, HAMA and PANSS scores all exceeded 0.8. In addition, they were blinded to the patient's clinical status.

### 2.3 Blood samples and biochemical indictors

Serum samples were collected from all subjects between 8 and 9 a.m. after an overnight fast and immediately sent to the hospital's laboratory center for analysis by 11 a.m. on the same day. Fasting biochemical parameters were measured, including TSH, free triiodothyronine (FT3), free thyroxine (FT4), anti‐thyroglobulin (TgAb), and thyroid peroxidase antibody (TPOAb), triglycerides (TG), total cholesterol (TC), high‐density lipoprotein cholesterol (HDL‐C), low‐density lipoprotein cholesterol (LDL‐C), and fasting blood glucose (FBG). The patients' height and weight, as well as systolic blood pressure (SBP) and diastolic blood pressure (DBP), were also recorded.

SCH was defined as a combination of elevated TSH and normal FT4 levels (Li et al., 2017). The normal range for TSH was 0.27–4.2 mIU/L, FT3 was 3.10–6.8 pmol/L, and FT4 was 10–23 pmol/L. In this study, SCH was diagnosed when TSH levels exceeded 4.2 mIU/L, while FT4 levels remained within the reference range (Li, 2018).

### 2.4 Statistical analysis

The Kolmogorow–Smirnov test was applied to examine the normality of the data. Normally distributed continuous variables were presented as means and standard deviations (SD); skewed quantitative variables were expressed as median (interquartile range, IQR) and categorical variables as frequencies and percentages. One‐way analysis of variance (ANOVA) was used for the comparison of normally distributed continuous variables, and Mann–Whitney *U* test was used for nonnormally distributed variables. Chi‐square tests were performed to compare categorical variables. To examine the correlates of suicide attempts, univariate analyses were conducted among the whole MDD patients with SCH as well as for male and female patients individually. Variables that showed statistical significance and clinical relevance were incorporated into a binary logistic regression model (Backward: Wald). Bonferroni correction was used to adjust for multiple testing. IBM SPSS Statistics, version 20.0 for Windows, was employed for all analyses. The reported *p* values were two‐sided, and statistical significance was defined as *p *< .05.

## 3 RESULTS

### 3.1 Sex differences in prevalence of suicide attempts among MDD patients with SCH

**FIGURE 1:**
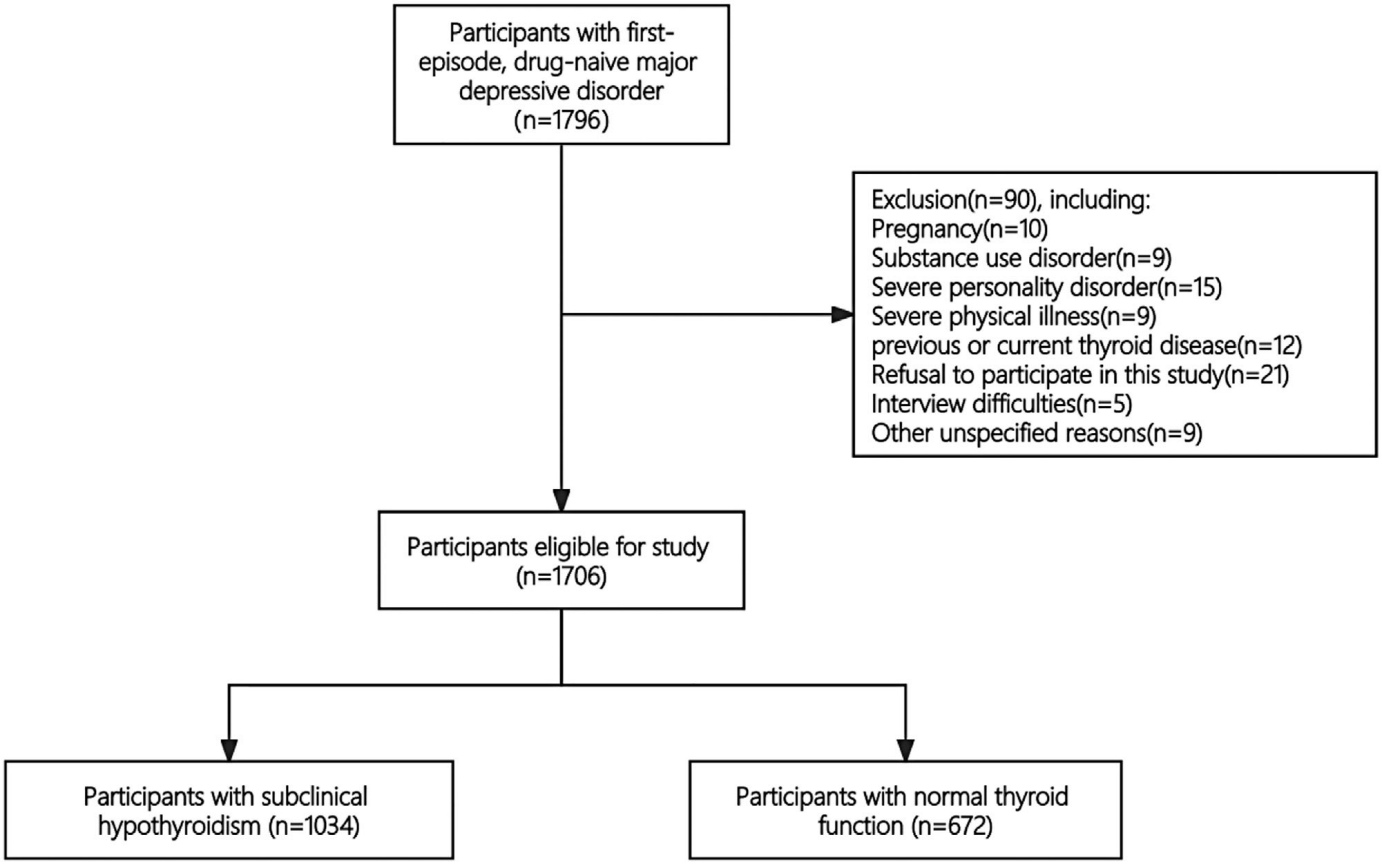
Flow diagram of the screening and enrollment of study participants.

A total of 1706 patients with FEDN MDD were included in this study, including 1034 MDD patients with comorbid SCH. The study's flowchart is presented in Figure 1. The proportion of MDD patients with SCH was 60.2% (1034/1718). Demographic characteristics for MDD patients, with and without SCH, are outlined in Table S1. Suicide attempt rates were notably higher in MDD patients with SCH (25.4%, 263/1034) compared to those without SCH (12.2%, 82/672) (*χ* ^2^*^^* = 44.21, OR = 2.4, *p *< .001). After adjusting for HAMA and HAMD scores, it was found that MDD patients with SCH had a 67% increased risk of suicide attempts compared to those without SCH (OR = 1.67, 95% CI = 1.22‐2.27, *p *= .001).

In MDD patients with SCH, the rate of suicide attempts was 24.7% (88/356) among male patients and 25.8% (175/678) among female patients, with no significant difference observed between the sexes (*χ* ^2 ^= 0.15, *p* = .76) (shown in Table 1).

**TABLE 1:** . Sex differences in demographic, clinical, and biochemical indicators among MDD patients with SCH.

Variables	Male (*n *= 356)	Female (*n *= 678)	*F/Z/χ^2^*	*p*
Age, years (median (IQR))	32 (22)	38 (23)	4.01	<.001
Age of onset, years (median (IQR))	32 (21)	37.5 (22)	4.04	<.001
Education (*n*, %) Junior high school High school Bachelor's degree Master's degree	74 (20.8%) 163 (45.8%) 105 (29.5%) 14 (3.9%)	54 (20.1%) 125 (46.6%) 80 (29.9%) 9 (3.4%)	1.53	.13
Marital status (single/married)	113/243	169/509	5.47	.02^a^
Duration of illness, months (median (IQR))	6 (6)	6 (5)	1.60	.11
HAMD (M ± SD)	31.27 ± 2.76	31.22 ± 2.69	0.08	.78
HAMA (M ± SD)	20.98 ± 3.58	21.32 ± 3.67	2.04	.15
Suicide attempts (*n*, %)	88 (24.7%)	175 (25.8%)	0.15	.76
Psychotic positive score, mean ± SD	9.2 ± 4.6	9.7 ± 5.1	2.19	.14
TSH, mIU/L (M ± SD)	6.54 ± 1.67	6.76 ± 1.95	3.19	.07
FT4, pmol/L (M ± SD)	16.77 ± 3.03	16.55 ± 3.01	1.22	.27
FT3, pmol/L (M ± SD)	4.96 ± 0.73	4.91 ± 0.72	1.26	.26
TgAb, IU/L (median (IQR))	22.20 (47.24)	23.12 (76.77)	1.31	.19
TPOAb, IU/L (median (IQR))	19.83 (34.19)	22.08 (43.95)	0.09	.93
FBG, mmol/L (M ± SD)	5.54 ± 0.63	5.63 ± 0.63	4.22	.04^a^
TC, mmol/L (M ± SD)	5.58 ± 1.05	5.68 ± 1.05	1.74	.19
TG, mmol/L (M ± SD)	2.20 ± 0.98	2.27 ± 0.97	1.09	.30
HDL‐C, mmol/L (M ± SD)	1.17 ± 0.32	1.15 ± 0.29	1.26	.26
LDL‐C, mmol/L (M ± SD)	3.20 ± 0.85	3.22 ± 0.88	0.22	.64
BMI, kg/m^2^ (M ± SD)	24.70 ± 2.19	24.59 ± 1.86	0.78	.38
Systolic BP, mmHg (M ± SD)	122.39 ± 9.07	123.92 ± 9.19	6.52	.01^a^
Diastolic BP, mmHg (M ± SD)	77.72 ± 6.62	77.64 ± 6.29	0.03	.86

Abbreviation: MDD, major depressive disorder; SCH, subclinical hypothyroidism; HAMD, Hamilton Depression Scale; HAMA, Hamilton Anxiety Scale; TSH, thyroid stimulating hormone; FT4, free thyroxine; FT3, free triiodothyronine; TgAb, anti‐thyroglobulin; TPOAb, thyroid peroxidase antibody; FBG, fasting blood glucose; TC, total cholesterol; TG, triglycerides; HDL‐C, high‐density lipoprotein cholesterol; LDL‐C, low‐density lipoprotein cholesterol; BMI, body mass index; BP, blood pressure; IQR, interquartile range; M ± SD, mean ± standard deviation.

^a^The *p* values did not pass the Bonferroni correction (Bonferroni corrected *p* < .05/22 = .002).

### 3.2 Sex differences in demographic, clinical, and biochemical indicators among MDD patients with SCH

As depicted in Table 1, female patients exhibited older age, a higher likelihood of being married, and a later age of onset compared to male MDD patients with SCH (all *p *< .001). Although SBP (*p* = .01) and FBG (*p* = .04) were elevated in female patients compared to males, these differences did not meet the threshold for significance after Bonferroni correction (Bonferroni corrected *p *< .05/22 = .002). Notably, there were no statistically significant sex differences observed in education levels, illness duration, HAMD, HAMA and psychotic positive score, or other biological indicators (all *p* > .05).

### 3.3 Demographic, clinical, and biochemical indicators in MDD patients comorbid SCH with and without suicide attempts

As shown in Table 2, MDD patients combined with SCH who attempted suicide exhibited significantly higher scores in HAMD (*p *< .001), HAMA (*p *< .001), PANSS positive symptoms (*p *< .001), as well as elevated levels of TSH (*p *< .001), TPOAb (*p *< .001), TgAb (*p *< .001), FBG (*p *< .001), TC (*p *< .001), TG (*p *< .001), HDL‐C (*p *= .004), LDL‐C (*p *< .001), SBP (*p *< .001), and DBP (*p *< .001) compared to those without suicide attempts. However, HDL‐C levels did not reach significance after Bonferroni correction (Bonferroni corrected *p *< .05/22 = 0.002).

**TABLE 2:** Clinical and biochemical characteristics of MDD patients with SCH with and without suicide attempts.

Variables	Nonsuicide MDD with SCH (*n* = 771)	suicide MDD with SCH (*n* = 263)	*F/Z/χ* ^2^	*p*
Sex (male/female)	268/503	88/175	0.147	.70
Age, years (median (IQR))	35.0 (22)	35.0 (22)	0.652	.41
Age of onset, years (median (IQR))	35.0 (22)	35.0 (22)	0.641	.42
Education (*n*, %)			3.19	.36
Junior high school	184 (23.9)	75 (28.5)		
High school	340 (44.1)	109 (41.4)		
Bachelor's degree	203 (26.3)	61 (23.2)		
Master's degree	44 (5.7)	18 (6.8)		
Duration of illness, months (median (IQR))	6.0 (5.5)	6.0 (5.5)	0.721	.29
Marital status (single/married)	207/564	75/188	0.275	.60
HAMD, mean ± SD	30.72 ± 2.54	32.76 ± 2.63	123.657	< .001
HAMA, mean ± SD	20.29 ± 3.17	23.87 ± 3.62	232.777	< .001
Psychotic positive score, mean ± SD	8.49 ± 3.64	12.42 ± 6.82	139.696	< .001
TSH, mIU/L (M ± SD)	6.24 ± 1.57	7.96 ± 2.06	198.167	< .001
FT4, pmol/L (M ± SD)	21.6 (31.6)	40.4 (177.4)	59.857	< .001
FT3, pmol/L (M ± SD)	18.6 (24.1)	36.7 (242)	59.044	< .001
TgAb, IU/L (median (IQR))	4.92 ± 0.73	4.95 ± 0.71	0.296	.587
TPOAb, IU/L (median (IQR))	16.61 ± 3.00	16.67 ± 3.09	0.082	.775
FBG, mmol/L (M ± SD)	5.54 ± 0.60	5.76 ± 0.69	22.717	< .001
TC, mmol/L (M ± SD)	5.50 ± 1.01	6.05 ± 1.05	56.334	< .001
TG, mmol/L (M ± SD)	1.19 ± 0.31	1.08 ± 0.27	25.244	< .001
HDL‐C, mmol/L (M ± SD)	2.19 ± 0.95	2.39 ± 1.03	8.195	.004^a^
LDL‐C, mmol/L (M ± SD)	3.14 ± 0.86	3.42 ± 0.86	20.667	< .001
BMI, kg/m^2^ (M ± SD)	24.70 ± 1.80	24.43 ± 2.43	3.667	.056
Systolic BP, mmHg (M ± SD)	122.21 ± 8.16	126.85 ± 10.93	52.681	< .001
Diastolic BP, mmHg (M ± SD)	76.98 ± 5.78	79.70 ± 7.61	36.807	< .001

Abbreviations: MDD, major depressive disorder; SCH, subclinical hypothyroidism; HAMD, Hamilton Depression Scale; HAMA, Hamilton Anxiety Scale; TSH, thyroid stimulating hormone; FT4, free thyroxine; FT3, free triiodothyronine; TgAb, anti‐thyroglobulin; TPOAb, thyroid peroxidase antibody; FBG, fasting blood glucose; TC, total cholesterol; TG, triglycerides; HDL‐C, high‐density lipoprotein cholesterol; LDL‐C, low‐density lipoprotein cholesterol; BMI, body mass index; BP, blood pressure; IQR, interquartile range; M ± SD, mean ± standard deviation.

^a^The *p* values did not pass the Bonferroni correction (Bonferroni corrected *p* < .05/22 = .002).

### 3.4 Sex differences in correlates of suicide attempts in MDD patients with SCH

Risk factors associated with suicide attempts were examined through binary logistic regression. Variables demonstrating significant differences in univariate analysis or deemed clinically relevant were included in the logistic regression (Backward: Wald) to identify risk factors for suicide attempts within the entire group as well as separately for female and male patients. As demonstrated in Table 3, HAMD score (OR = 1.11, 95% CI 1.02‐1.21), HAMA score (OR = 1.20, 95% CI 1.12‐1.28), TSH (OR = 1.25, 95% CI 1.12‐1.39), TPOAb (OR = 1.002, 95% CI 1.001‐1.003), and SBP (OR = 1.04, 95% CI 1.02‐1.06) were independently associated with suicide attempts among MDD patients with SCH. Within male patients (Table 4), the factors included HAMA score (OR = 1.24, 95% CI 1.13‐1.36), TSH (OR = 1.30, 95% CI 1.09‐1.57), TPOAb (OR = 1.003, 95% CI 1.002‐1.005), and LDL‐C (OR = 1.50, 95% CI 1.06‐2.13). For female patients (Table 5), risk factors encompassed HAMD score (OR = 1.17, 95% CI 1.06‐1.31), HAMA score (OR = 1.19, 95% CI 1.10‐1.29), TSH (OR = 1.25, 95% CI 1.10‐1.41), TPOAb (OR = 1.002, 95% CI 1.001‐1.003), and SBP (OR = 1.04, 95% CI 1.02‐1.07).

**TABLE 3:** Binary logistic regression evaluating the correlates of suicide attempts in MDD patients with SCH.

Variables	*B*	Wald	*p*	OR	95% CI
HAMD	0.11	6.85	.009	1.11	1.02–1.21
HAMA	0.18	30.01	<.001	1.20	1.12–1.28
TSH	0.22	17.91	<.001	1.25	1.12–1.39
TPOAb	0.002	26.41	<.001	1.002	1.001–1.003
Systolic BP	0.04	11.86	.002	1.04	1.02–1.06

Abbreviations: MDD, major depressive disorder; SCH, subclinical hypothyroidism; HAMD, Hamilton Depression Scale; HAMA, Hamilton Anxiety Scale; TSH, thyroid stimulating hormone; TPOAb, thyroid peroxidase antibody; BP, blood pressure.

**TABLE 4:** Binary logistic regression evaluating the correlates of suicide attempts in male MDD patients with SCH.

Variables	*B*	Wald	*p*	OR	95% CI
HAMA	0.22	22.86	<.001	1.24	1.13–1.36
TSH	0.27	8.10	.004	1.30	1.09–1.57
TPOAb	0.003	16.95	<.001	1.003	1.002–1.005
LDL‐C	0.41	5.23	.02	1.50	1.06–2.13

Abbreviations: SCH, subclinical hypothyroidism; HAMA, Hamilton Anxiety Scale; TSH, thyroid stimulating hormone; TPOAb, thyroid peroxidase antibody; LDL‐C, low‐density lipoprotein cholesterol.

**TABLE 5:** Binary logistic regression evaluating the correlates of suicide attempts in female MDD patients with SCH.

Variables	*B*	Wald	*p*	OR	95% CI
HAMD	0.16	8.93	.003	1.17	1.06–1.31
HAMA	0.17	17.65	<.001	1.19	1.10–1.29
TSH	0.22	12.38	<.001	1.25	1.10–1.41
TPOAb	0.002	13.18	<.001	1.002	1.001–1.003
Systolic BP	0.04	9.96	.002	1.04	1.02–1.07

Abbreviations: MDD, major depressive disorder; SCH, subclinical hypothyroidism; HAMD, Hamilton Depression Scale; HAMA, Hamilton Anxiety Scale; TSH, thyroid stimulating hormone; TPOAb, thyroid peroxidase antibody; BP, blood pressure.

## 4 DISCUSSION

To the best of our knowledge, this study is the first study to explore the sex differences in prevalence and related factors of suicide attempts among Chinese Han FEDN MDD patients with comorbid SCH. The findings from this study indicate: (1) a 35.4% prevalence of suicide attempts in MDD patients with SCH, significantly higher than the 12.2% among those without SCH; (2) no discernible sex difference in the prevalence of suicide attempts among MDD patients comorbid SCH; and (3) elevated HAMA score, TSH and TPOAb levels were associated with heightened risk for suicide attempts in both male and female MDD patients comorbid SCH. Additionally, LDL‐C demonstrated a significant association with increased suicide risk exclusively in male patients, while HAMD score and SBP showed notable associations exclusively in female patients.

In our study, we observed a significantly higher rate of suicide attempts among MDD patients with SCH compared to those without SCH, supporting the notion that thyroid dysfunction escalates suicide risk in individuals with depression (Sanna et al., 2014; Toloza et al., 2021). This suggests a considerable impact of thyroid function on suicide among depressed individuals, emphasizing the need for heightened attention and early intervention for these patients. Interestingly, this study did not identify any sex differences in the incidence of suicide attempts among MDD patients with comorbid SCH. To the best of our knowledge, our investigation is the first to explore sex differences in suicide attempts prevalence among MDD individuals comorbid SCH. Prior research has indicated that suicide attempts were notably more common among females, whereas completed suicides were significantly more prevalent among males (Miranda‐Mendizabal et al., 2019). Cultural variations contribute to sex differences in suicide. For instance, a psychological autopsy study in South Tyrol revealed a higher suicide risk among men compared to women (Giupponi et al., 2016). However, a psychological autopsy study in China reported no sex difference in suicide risk (Phillips et al., 2002). Societal changes also influence sex‐based distinctions in suicide. Before 2000, females in China had a notably higher rate of completed suicides than males. However, after 2000, this sex difference diminished (Li et al., 2012). Considering these disparities, comprehensive large‐scale, multinational, and multiethnic longitudinal studies are crucial for a thorough understanding of the sex differences in suicide attempts among MDD patients with comorbid SCH.

Although numerous studies have highlighted the link between thyroid function and depression (Ojha et al., 2013; Zhao et al., 2018), the evidence regarding whether thyroid dysfunction escalates the risk of suicidal behavior in MDD patients remains inconclusive. Our study indicated a positive association between TSH and TPOAb levels and suicide attempts in both male and female patients. This aligns with some earlier research, which reported that higher serum TSH level projected previous suicide attempts (Berlin et al., 1999; Zhang et al., 2022). However, some studies have suggested contrasting findings, showing that MDD patients who attempted suicide had lower TSH levels compared to nonattempters (Duval et al., 2010; Peng et al., 2018). Our previous study showed that elevated TSH levels were associated with increased moderate to severe anxiety symptoms in young MDD patients with SCH (Yang et al., 2022). Anxiety symptoms may increase the risk of suicide (Lin et al., 2014; Yang et al., 2019). Therefore, elevated TSH levels may potentially increase suicide risk by increasing anxiety symptoms in MDD patients. The association between TSH level and suicide attempts in MDD patients may stem from the interplay neurotransmitters, hypothalamic‐pituitary‐adrenal (HPA) axis and HPT axis. First, studies have demonstrated that reduced 5‐hydroxytryptamine (5‐HT) transporter binding in specific brain regions is linked to suicidal thoughts (Currier & Mann, 2008; Mann, 2013). Additionally, evidence from animal studies strongly suggests that the HPT axis significantly influences 5‐HT neurotransmission in the adult brain (Bauer et al., 2002). Secondly, several lines of evidence suggested a dysregulation of the HPA axis stress response activity in suicidal patients (O'Connor et al., 2020; Tsigos & Chrousos, 2002). Prolonged stress responses in the HPA axis could hinder HPT function, resulting in decreased FT4 and subsequently increased TSH levels (Duval et al., 2002). Elevated TPOAb levels often signal autoimmune thyroid disease as a contributing factor to hypothyroidism (Vasikaran & Loh, 2021). Consistent with our findings, previous studies have revealed a substantial association between elevated TPOAb levels and depression, suggesting that MDD patients with elevated TPOAb levels experience more severe symptoms of depression and anxiety, potentially heightening the likelihood of suicide attempts (Carta et al., 2004; Degner et al., 2015). Heiberg Brix et al. discovered an increased incidence of suicide among patients with Hashimoto's thyroiditis (Heiberg Brix et al., 2019). The dysregulation of the immune system has long been implicated in the pathophysiology of suicidality (Benros et al., 2013; Lund‐Sørensen et al., 2016; Sudol & Mann, 2017). It was found that individuals who experience immune activation are more likely to engage in suicide attempts. This is believed to be linked to higher levels of neurotoxicity caused by inflammation and nitro‐oxidative stress (Vasupanrajit et al., 2021).

In our study, among female MDD patients with comorbid SCH, both depression and anxiety heightened the risk of suicide attempts, while among male patients, suicide attempts were solely linked to anxiety. A meta‐analysis exploring suicide risk factors among individuals with MDD highlighted severe anxiety and depression as linked to increased suicide risk (Hawton et al., 2013). Another study focusing on Chinese university students identified depression, anxiety, stress, and hopelessness as primary risk factors for student suicide (Lew et al., 2019). Parker and Brotchie's research identified higher‐order biological factors, such as neuroticism, stress responsiveness, or limbic system hyperactivity, contributing significantly to sex‐specific differences in depression and anxiety manifestations (Parker & Brotchie, 2010). Women tend to internalize problems, leading to a downward spiral of low self‐esteem and depression, while men may display depression through aggressive behavior, low impulse control, substance abuse, irritability, restlessness and suicidality (Angst et al., 2002). These sex differences in the relationship between depression, anxiety, and suicide may be due to a combination of biological structures and social roles (Harlow et al., 2004; Parker & Brotchie, 2010; Swetlitz, 2021).

In this study, we discovered that SBP emerged as an independent factor for suicide attempts among female MDD patients with comorbid SCH, but not in male patients. Notably, there is an elevated incidence of hypertension among individuals with depression (Adamis & Ball, 2000). The physiological mechanisms between depression and blood pressure may involve the influence of the sympathetic nervous system (Townsend et al., 1998). An interaction exists between depression, anxiety, and hypertension, where anxiety and depression increase the risk of suicide attempts, and hypertension acts as a clinical vulnerability factor for suicidal ideation (Lehmann et al., 2018). A study in China reported a 19.6% prevalence of suicidal ideation among hypertensive patients (Ge et al., 2019). Previous research indicated that hypertension and psychological stress work together to form a vicious cycle leading to occasional suicidal thoughts (Yusuf et al., 2004). This sex discrepancy might stem from various factors. Firstly, the molecular, cellular and tissue levels that regulate arterial blood pressure vary between men and women (Colafella & Denton, 2018). Secondly, psychosocial factors associated with suicide seem to be more closely related to hypertension in female patients. In the INTERHEART study, women showed a notably higher risk of myocardial infarction attributable to psychosocial factors compared to men, suggesting that women with cardiovascular disease might be more susceptible to such influences (Yusuf et al., 2004).

Our study revealed that elevated LDL‐C levels were associated with an increased risk of suicide attempts specifically in male MDD patients with SCH, whereas no such association was observed in female patients. Multiple studies suggest an association between suicidal behavior and low levels of TC, HDL‐C, and LDL‐C in MDD patients (Ainiyet & Rybakowski, 2014; Lester, 2002; Maes et al., 1997; Muldoon et al., 1990). Conversely, Baek et al. (2014) found higher levels of TC, LDL‐C, and TG in MDD patients with suicidal tendencies. These inconsistencies may be due to different demographic variables. Previous studies have also revealed sex‐specific associations between lipid levels and suicide or depression. For instance, Golier et al. (1995) identified an association between cholesterol levels and suicide attempts solely in males. Similarly, a study in Korea identified high TC levels as a risk factor for depression in men, but not in women (Lee et al., 2022; Lee et al., 2022). Moreover, a meta‐analysis noted a positive association between serum LDL‐C levels and depression risk (Persons & Fiedorowicz, 2016). Therefore, high LDL‐C levels might potentially elevate suicide attempts by increasing the risk of depression. The main neurobiological mechanism explaining the link between low cholesterol and suicidal behavior is the cholesterol‐serotonin impulse model (Engelberg, 1992). Research has demonstrated that reduced membrane cholesterol negatively impacts the serotonin transporter function (Scanlon et al., 2001). Additionally, diminished serotonin transporter binding in specific brain regions has been linked to suicidal ideation (Currier & Mann, 2008; Mann, 2013). The mechanisms underlying the sex differences in the association between serum lipids and suicide attempts remain unclear and warrant further investigation.

This study has several limitations. Firstly, due to the inherent constraints of cross‐sectional studies, establishing a causal relationship between correlates and suicide attempts is challenging. Secondly, the collection of suicidal information relied on semistructured interviews with patients, family members, and clinicians rather than employing a standardized assessment scale like the Beck Suicidal Ideation Scale (BSSI), potentially impacting result validity. Future research should aim to address this gap. Thirdly, factors that were not measured or are unknown might have residual confounding effects, and these could not be entirely excluded. Fourthly, the study's participants were exclusively from the Han Chinese population and recruited from outpatient clinics, thereby limiting the generalizability of the study's findings. Considering the limitations of this study, the findings should be cautiously interpreted and validated by future longitudinal studies encompassing a broader spectrum of factors.

## 5 CONCLUSION

In summary, our findings indicate a higher prevalence of suicide attempts among FEDN MDD patients with SCH compared to those without SCH. Within the FEDN MDD patients with SCH, no significant sex differences were observed in the prevalence of suicide attempts. Elevated levels of TSH and TPOAb were identified as risk factors for suicide attempts among both male and female MDD patients with SCH. LDL‐C levels exhibited an association solely in male patients, while SBP showed such an association exclusively in female patients. Notably, depression and anxiety emerged as risk factors for suicide attempts in female patients, while in male patients, anxiety alone appeared to contribute to such attempts. These findings underscore the critical need for comprehensive assessment and targeted management of suicide risk among depressed population with comorbid SCH, emphasizing the importance of considering sex differences in this high‐risk population.

## AUTHOR CONTRIBUTIONS

**Xue Tian**: Formal analysis; investigation; methodology; validation; visualization; writing—original draft. **Xiao‐en Liu**: Formal analysis; methodology; validation. **Fengfeng Bai**: Investigation; validation; visualization. **Meijuan Li**: Formal analysis; validation; writing—original draft. **Yuying Qiu**: Formal analysis; methodology. **Qingyan Jiao**: Methodology. **Jie Lie**: Funding acquisition; validation; writing—review and editing. **Xiang‐Yang Zhang**: Data curation; funding acquisition; methodology; project administration; resources; validation; writing—review and editing.

## CONFLICT OF INTEREST STATEMENT

No conflicts of interest.

### PEER REVIEW

The peer review history for this article is available at https://publons.com/publon/10.1002/brb3.3578.

## Supporting information


**Table S1**. Demographics, clinical characteristics, and biochemical parameters between MDD patients with and without SCH.

## Data Availability

The datasets used during the current study are available from the corresponding author on reasonable request. The data are not publicly available due to privacy or ethical restrictions.
