# Supplementary material for: Sex differences in correlates of suicide attempts in Chinese Han first‐episode and drug‐naïve major depressive disorder with comorbid subclinical hypothyroidism: A cross‐sectional study
Source: Brain Behav. 2024 Jun 6;14(6):e3578. doi: 10.1002/brb3.3578 (PMC11156525; doi:10.1002/brb3.3578)
Supplement: Supplementary file 1 — Table S1. Demographics, clinical characteristics, and biochemical parameters between MDD patients with and without SCH. [file BRB3-14-e3578-s001.docx]

**Table S1.** Demographics, clinical characteristics and biochemical parameters between MDD patients with and without SCH

| Variables | Patients with SCH(*n*=1034) | Patients without SCH(*n*=672) | *F/Z/χ2* | *p* |
| --- | --- | --- | --- | --- |
| Sex (Male/Female) | 356/678 | 230/442 | 0.007 | 0.93 |
| Age, years, (median (IQR)) | 35(22) | 34(22) | 3.47 | < 0.01 |
| Age of onset, years, (median (IQR)) | 35(22) | 33(22) | 3.42 | < 0.01 |
| Education (*n*, %)  Junior high school  High school  Bachelor’s degree  Master’s degree | 259(25%)  449(43.4%)  264(25.5%)  62(6%) | 148(22%)  309(46%)  182(27.1%)  33(4.9%) | 3.40 | 0.33 |
| Marital status(single/married) | 282/752 | 218/454 | 5.25 | 0.02^a^ |
| Duration of illness, months, (median (IQR)) | 6(5.5) | 5(5) | 9.82 | **<0.001** |
| HAMD (M±SD) | 31.24±2.72 | 28.85±2.7 | 315.47 | **<0.001** |
| HAMA (M±SD) | 21.20±3.64 | 20.21±3.12 | 33.90 | **<0.001** |
| Suicide attempts (*n*, %) | 263(25.4%) | 82(12.2%) | 44.21 | **<0.001** |
| Psychotic positive score, Mean ± SD | 7.90±3.15 | 9.49±4.96 | 28.50 | < 0.01 |
| TSH, mIU/L, (M±SD) | 6.68±1.86 | 2.58±1.07 | 2679.34 | **<0.001** |
| FT4, pmol/L, (M±SD) | 16.63±3.02 | 16.80±3.07 | 1.37 | 0.24 |
| FT3, pmol/L, (M±SD) | 4.93±0.73 | 4.88±0.70 | 2.13 | 0.15 |
| TgAb, IU/L, (median (IQR)) | 22.64(72.31) | 21.42(28.34) | 7.13 | **<0.001** |
| TPOAb, IU/L, (median (IQR)) | 21.41(42.96) | 17.43(22.14) | 8.28 | **<0.001** |
| FBG, mmol/L, (M±SD) | 5.60±0.63 | 5.09±0.55 | 290.89 | **<0.001** |
| TC, mmol/L, (M±SD) | 5.64±1.05 | 4.64±0.90 | 415.79 | **<0.001** |
| TG, mmol/L, (M±SD) | 2.24±0.98 | 2.06±0.99 | 14.45 | **<0.001** |
| HDL-C, mmol/L, (M±SD) | 1.16±0.30 | 1.31±0.23 | 125.33 | **<0.001** |
| LDL-C, mmol/L, (M±SD) | 3.21±0.87 | 2.63±0.72 | 213.11 | **<0.001** |
| BMI, kg/m2, (M±SD) | 24.63±1.98 | 23.97±1.75 | 48.68 | **<0.001** |
| Systolic BP, mmHg, (M±SD) | 123.39±9.17 | 113.35±10.61 | 430.40 | **<0.001** |
| Diastolic BP, mmHg, (M±SD) | 77.67±6.40 | 73.26±6.40 | 193.21 | **<0.001** |

Abbreviations: *MDD* major depressive disorder, *SCH* subclinical hypothyroidism, *HAMD* Hamilton Depression Scale, *HAMA* Hamilton Anxiety Scale, *TSH* thyroid stimulating hormone, *FT4* free thyroxine, *FT3* free triiodothyronine, *TgAb* anti-thyroglobulin, *TPOAb* thyroid peroxidase antibody, *FBG* fasting blood glucose, *TC* total cholesterol, *TG* triglycerides, *HDL-C* high-density lipoprotein cholesterol, *LDL-C* low-density lipoprotein cholesterol, *BMI* Body Mass Index, *BP* blood pressure, *IQR* interquartile range, *M±SD* mean±standard deviation

Note: ^a^ The *p* values did not pass the Bonferroni correction (Bonferroni corrected p<0.05/23=0.002).
